# Pregnancy and breast cancer in young women: current updates and future directions

**DOI:** 10.1177/17588359251369973

**Published:** 2025-09-06

**Authors:** Shiliang Zhang, Sena B. Guclu, Marla Lipsyc-Sharf, Nimmi S. Kapoor

**Affiliations:** Division of Hematology and Oncology, Department of Medicine, University of California, Los Angeles, Los Angeles, CA, USA; School of Medicine, Koç University, Istanbul, Turkey; Division of Hematology and Oncology, Department of Medicine, University of California, Los Angeles, Los Angeles, CA, USA; Division of Surgical Oncology, Department of Surgery, University of California, Los Angeles, 15503 Ventura Blvd, Suite 150, Los Angeles, CA 90095, USA

**Keywords:** breast cancer, chemotherapy, hormone therapy, postpartum, pregnancy, targeted therapy

## Abstract

The relationship between pregnancy and breast cancer is complicated. On one hand, pregnancy can influence breast cancer risk and tumor biology, and on the other, a breast cancer diagnosis and its subsequent management can significantly affect fertility, family planning, and future pregnancies. This interaction presents challenges unique to young women with breast cancer (YWBC). This review provides an updated overview of YWBC and pregnancy-associated breast cancer (PABC), including the rising prevalence of YWBC, current understanding of the biology of PABC and its subtypes, and the management of PABC. We address the critical issue of how breast cancer treatment impacts family planning and future pregnancies, including current data on interrupting endocrine treatment for pregnancy. Additionally, we examine recent advancements in early-stage breast cancer as well as gaps in knowledge regarding how targeted treatments affect ovarian reserve. Finally, we highlight key areas of future research that could improve the prevention, management, and understanding of cancer treatment’s impact on fertility in YWBC.

## Introduction

While pregnancy is associated with long-term protective effects against breast cancer, it initially induces an increased risk of breast cancer development in the postpartum setting.^1^ Large epidemiological studies have demonstrated that while pregnancy ultimately decreases breast cancer risk, this benefit is primarily observed in younger women less than 35 years old.^2^ Conversely, women experiencing their first childbirth at an advanced age face an elevated breast cancer risk.^2,3^ This relationship has become increasingly relevant as the average age at first childbirth has been rising in many countries.^4,5^ As a result of this trend, there is a higher risk of breast cancer associated with pregnancy conferred to women who fall into the category of young women with breast cancer (YWBC), typically defined as those diagnosed before 40–45 years of age. While the incidence of breast cancer diagnosis in the US has overall stabilized, driven in large part by patients 50 years or older at diagnosis, multiple cross-sectional analyses of SEER data indicate the incidence of breast cancer is increasing in younger women.^6,7^ Both YWBC and breast cancer occurring after pregnancy tend to exhibit more aggressive features, including larger tumors, higher stages at diagnosis, and a greater likelihood of lymph node involvement.^8–11^ These cancers are also more frequently associated with unfavorable subtypes such as triple-negative or HER2-positive disease.^3,12^ The rising trends in delayed childbearing and YWBC incidence, coupled with the more aggressive nature of these breast cancers, underscore the importance of understanding and addressing the complex relationship between pregnancy and breast cancer risk in younger women. Furthermore, the unique challenges seen with YWBC and particularly pregnancy-associated breast cancer (PABC) present opportunities for research and treatment. This commentary will provide an update on YWBC and PABC, exploring current understanding of biology, risk factors, management, and future research directions.

## Methods

We performed a focused literature review using the PubMed database to identify relevant studies focused on pregnancy-related breast cancer (PrBC), postpartum breast cancer (PPBC), and the management of breast cancer in young women in these contexts. Search terms included a combination of Medical Subject Headings (MeSH) terms and free-text terms, such as: (“pregnancy associated” OR “pregnancy related” OR “postpartum”) AND (breast cancer (MeSH Terms)). We also included variations of these terms with and without hyphens to ensure comprehensive retrieval. The search covered a 20-year period from January 1, 2005 to December 31, 2024. For this review, we prioritized studies published within the last 5 years—January 1, 2020, to December 31, 2024—to emphasize the most current evidence. Two authors independently screened all titles and abstracts for relevance. Eligible studies included peer-reviewed publications, such as original research, comprehensive reviews, and clinical guidelines. We excluded articles published in languages other than English, as well as non-peer-reviewed publications, including case reports, letters, and pre-prints.

## Biology of PABC and YWBC

**Figure 1.**
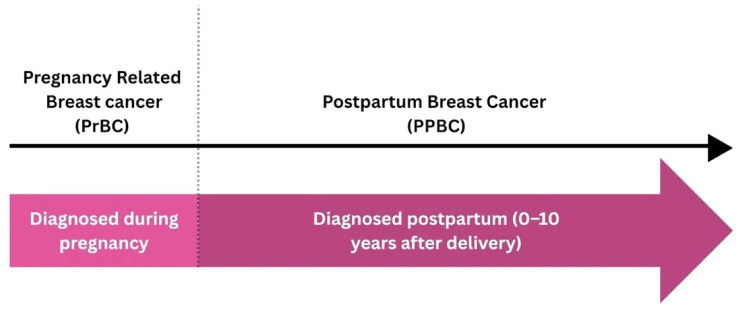
Timeline and definition of PABC (PrBC vs PPBC). PABC, pregnancy-associated breast cancer; PPBC, postpartum breast cancer; PrBC, pregnancy-related breast cancer.

YWBC and PABC exhibit distinct biological characteristics that contribute to their aggressive nature and poor prognosis. PABC, traditionally defined as breast cancer diagnosed during pregnancy or within 1 year postpartum, is now recognized as two separate entities: PrBC and PPBC. PrBC occurs during pregnancy, while PPBC is diagnosed within 10 years after childbirth^13^ (see Figure 1). The outcomes of PrBC remain controversial. While PrBC is often associated with higher-risk features such as larger tumor size, higher grade, increased lymph node involvement, and more aggressive subtypes, retrospective data suggest that overall outcomes may be comparable to breast cancers diagnosed outside of pregnancy.^14–16^ It is generally accepted that PPBC carries higher-risk features and is associated with higher rates of recurrence and mortality.^17^ Studies indicate that within a cohort of YWBC, PPBC patients are at increased risk for metastasis and, when compared to nulliparous YWBC patients, have significantly lower overall survival.^18,19^

The unique biology of PPBC likely underscores its more aggressive nature and association with worse survival outcomes. In the postpartum setting, breast tissue undergoes profound tissue remodeling linked to postpartum mammary gland involution, which occurs after lactation ceases.^20^ This physiological event shares many characteristics with wound healing, including increased immune cell influx, extracellular matrix remodeling, and lymphangiogenesis.^21^ The pro-inflammatory tissue microenvironment in PPBC tumors exhibits immune cell phenotypes characterized by T cell exhaustion and immune tolerance, reflective of an immunosuppressed state.^20,22^ Furthermore, gland involution involves remodeling of the mammary extracellular matrix, which can cause increased concentration of tumor-promoting growth factors and result in the loss of basement membrane barrier function.^3^ Involution also causes an expansion of lymphatic vasculature, and PPBC tumors are associated with a significant increase in lymphatic vessel density, which may underlie the higher risk for lymph node metastasis in PPBC patients.^3,23^ While lobular regression during involution typically completes within 12–18 months postpartum, leukocytes and macrophages contributing to a pro-inflammatory microenvironment persist in the mammary gland beyond this period.^21^ Furthermore, distinct gene expression signatures associated with PABC have been shown to persist for up to 10 years after childbirth.^24^ Overall, these distinct biologic features of PPBC create a pro-tumorigenic environment in which early subclinical disease may be in a more favorable environment for tumor dissemination at the time of mammary gland involution and beyond.^3^

Gene expression studies in PABC, particularly in the postpartum subgroup (PPBC), have identified molecular pathways associated with more aggressive tumor biology. Compared to non-PABCs, PABCs are more often characterized by basal-like subtypes and overexpress genes associated with cell proliferation, DNA damage, and alterations in the TP53 pathway.^25^ Furthermore, PPBC may have distinct genomic features within the broader PABC category. Compared to nulliparous YWBC, young women with PPBC have distinct genomic signatures, including increased cell cycle gene expression and reduced estrogen receptor (ER) signaling, even in ER-positive tumors.^20^ This suggests that ER-positive (ER+) PPBC may behave more like ER-negative disease in terms of downstream signaling pathways. When comparing PrBC and PPBC gene signatures, a study found that PPBC samples demonstrated higher immune infiltration scores.^26^ Notably, the immune infiltration score included elevated expression of the immune checkpoint gene *PDCD1/PD-1*. Cell-type profiling of PPBC samples demonstrated increased immune cell populations, including macrophages, regulatory T cells, B cells, and neutrophils. In a separate prospective cohort study evaluating breast cancer patients who were nulliparous compared to those with PPBC, patients with PPBC within 2 years postpartum had a higher trend in PD-L1 expression (*n* = 7/12, 58.3% vs *n* = 4/19, 21.1%, *p* = 0.056).^27^ Additionally, findings from a substudy of the prospective observational FLEX study—which evaluates MammaPrint, BluePrint, and full transcriptome sequencing—support the aggressive nature of PPBC. The study found that patients with PPBC within 5 years of childbirth had a higher prevalence of MammaPrint High 2 compared to nulliparous women (30.3% vs 11.9% *p* = <0.05). The study also identified increased immune-mediated gene signatures in this cohort.^28^ Although exploratory, these findings demonstrate distinct immunological features in PPBC, which may have therapeutic implications—particularly for the use of immune checkpoint inhibitors in this population.^20^

YWBC are known to have a higher prevalence of germline mutations predisposing to breast cancer, including BRCA1 and BRCA2 mutations. Given that PABC is associated with more aggressive features and high-risk gene expression profiles, understanding the intersection of genetic predisposition and PABC development is important. In an age-matched cohort study of PABC and non-PABC patients, a significantly higher percentage of PABC patients reported a family history of breast cancer compared to patients with non-PABC (52.17% vs 26.53%; *p* = 0.0124).^25^ A cohort study evaluating germline mutations in 20 women with PABC found that 35% of patients had pathogenic mutations predisposing to breast cancer,^29^ while in the general YWBC population, fewer (about 15%) have germline mutations associated with higher breast cancer risk.^30,31^ Interestingly, a cohort study of YWBC patients with pathologic BRCA mutations found that PPBC was an independent risk factor for worse survival when compared to nulliparous patients.^32^ While limited, these data raise the need for further research on whether germline mutations may increase susceptibility to PABC and influence clinical outcomes.

**Figure 2.**
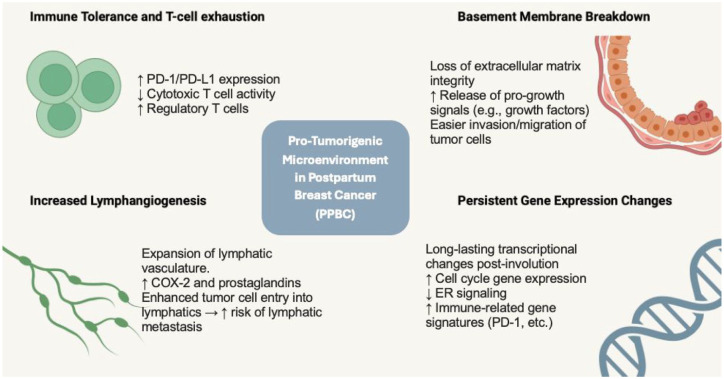
Biological pathways and tumor microenvironment in PPBC.^35^ PPBC, postpartum breast cancer.

Further research in molecular profiling is crucial to better understand the unique genomic and proteomic signatures of PPBC. These efforts may not only identify potential therapeutic targets, but also inform preventative strategies to decrease breast cancer risk in YWBC. Future research should focus on identifying risk factors to develop more precise methods for assessing breast cancer risk in postpartum women and implementing targeted screening strategies, particularly for those under 40 who do not undergo regular breast cancer screening. Postpartum breast involution is associated with significant inflammatory changes, and cyclooxygenase-2 (COX-2) has been implicated in involution-related lymphangiogenesis. Overexpression of COX-2 in postpartum breast tissue correlates with increased lymphatic vessel density, potentially through the elevated production of prostaglandins.^23^ In a preclinical study, postpartum mice exposed to celecoxib, a COX-2 inhibitor, were found to have a significant decrease in mammary lymphatic vessel density.^23^ Furthermore, COX-2 expression and its downstream product, prostaglandin E2, may contribute to a pro-tumorigenic immune environment.^33^ These findings have generated interest in exploring non-steroidal anti-inflammatory drugs (NSAIDs), such as ibuprofen, as potential agents to modulate the inflammatory milieu of the involuting breast and reduce the risk of PPBC. The forthcoming PREVENT PPBC I trial aims to evaluate the effect of NSAIDs on altering the immune and inflammatory characteristics of postpartum breast involution, with the goal of preventing PPBC development^34^ (see Figure 2). Understanding the distinct molecular pathways and tumor microenvironment of PPBC in YWBC is crucial for developing more effective treatments as well as prevention strategies to improve outcomes for YWBC.

## Current management of PABC

While treatment approaches for women with PPBC are similar to those for women with non-PPBC, managing breast cancer diagnosed during pregnancy (PrBC) presents unique challenges. In contrast, the management of PPBC generally follows standard treatment protocols for YWBC, including decisions around surgery, chemotherapy, endocrine therapy, and targeted therapies. However, the more aggressive clinical and pathological features seen in PPBC may influence treatment decisions.

Unlike PPBC, PrBC requires treatment decisions that balance maternal and fetal health. While breast cancer diagnosed during pregnancy is rare, reported at a rate of about 1 in 3000 pregnancies, it is the most common type of cancer diagnosed during pregnancy.^36,37^ The primary goal of managing PrBC is to maximize maternal survival outcomes while minimizing the risk of harm to the fetus. To achieve this, a multidisciplinary approach involving medical oncologists, surgical oncologists, obstetricians, and maternal-fetal medicine specialists is crucial for optimizing care. The careful calculation of risk-versus-benefit begins with diagnosis and staging when the clinician must consider the most appropriate imaging modality. In general, ionizing radiation exposure is avoided and minimized, given the concern for fetal malformations. In diagnosing and staging PrBC, ultrasound and non-contrast MRI are preferable to CT and PET/CT. However, the very small amount of radiation associated with mammograms and chest X-rays is generally considered safe if clinically necessary.^38,39^

Management of PrBC, like treatment of breast cancer in general, is determined by various factors such as stage, histological subtype, and patient comorbidities and preferences. However, in PrBC, there is an additional layer of complexity in considering the timing and modes of treatment to minimize risks to the fetus. Local treatment options for breast cancer include surgery and radiation. While surgery is generally considered safe during all trimesters of pregnancy, radiation is contraindicated in pregnancy due to the high risk for fetal malformations. Because radiation must be postponed until after delivery, the ability to pursue breast-conserving surgery is influenced by the expected time of radiation initiation. Therefore, more pregnant women with breast cancer diagnosed early in pregnancy may pursue mastectomy as a first line of treatment.^40^ If a woman is near term, breast-conserving surgery and radiation following delivery is a viable option. Many women with PrBC, however, will undergo neoadjuvant systemic therapy, and, given the young age and higher rate of genetic predisposition among women with PABC, we have observed that almost 50% of these women will choose bilateral mastectomy as their surgical treatment.^41^

Systemic treatments, including chemotherapy, HER2-targeted therapies, and endocrine therapies, have variable safety data during pregnancy. In the second and third trimesters, anthracycline-based regimens, such as doxorubicin, and alkylating agents, such as cyclophosphamide, are generally considered safe and have a comparatively longer history of safety data.^42,43^ In more recent years, taxanes, particularly paclitaxel, have also been shown to be safe in the second and third trimester.^44,45^ In contrast, endocrine therapy and HER2-targeted therapies are contraindicated during pregnancy. In studies summarizing results of patients exposed to tamoxifen during pregnancy, the rate of major malformations after tamoxifen exposure was 17.6% compared to 3% in the general non-exposed population.^46^ HER2-targeted agents are contraindicated during pregnancy due to obstetric risk, as trastuzumab has been associated with oligohydramnios and anhydramnios.^47^ There is a paucity of data regarding other HER2-targeted agents, such as pertuzumab, trastuzumab emtansine (T-DM1), and neratinib, which are increasingly used in the early breast cancer setting. MotHER (NCT00833963) is a prospective observational study including women with breast cancer exposed to trastuzumab, pertuzumab, or T-DM1 during pregnancy or within 7 months prior to conception (ClinicalTrials.gov identifier: NCT00833963).^48^ The pre-specified study accrual period for MotHER has concluded, and we anticipate that the results will be published in the near future. These findings may provide valuable insights into the safety and outcomes of HER2-targeted therapies in relation to pregnancy in breast cancer patients.

The increasing use of other targeted therapies in the routine treatment of early-stage breast cancer, including immunotherapy, CDK 4/6 inhibitors, and poly-ADP-ribose polymerase (PARP) inhibitors, presents a challenge regarding their safety in pregnancy. To our knowledge, there are no studies evaluating the safety of CDK 4/6 inhibitors or PARP inhibitors in pregnant women. These agents are contraindicated due to a lack of sufficient safety data, and the mechanism of these targeted therapies suggests potential risks in fetal development. CDK 4/6 inhibitors (palbociclib, ribociclib, abemaciclib) target cell cycle progression, and preclinical studies have demonstrated embryotoxicity.^49,50^ PARP inhibitors work by interfering with DNA repair mechanisms and have been shown to cause teratogenicity in animal studies.^51^ In addition, ongoing research is investigating the potential role of antibody drug conjugates (ADCs) other than T-DM1 for early-stage breast cancer. ADCs currently undergoing evaluation in the neoadjuvant and adjuvant settings for breast cancer treatment feature monoclonal antibodies—such as trastuzumab or a Trop-2 antibody—covalently linked to a cytotoxic payload. Given the association of trastuzumab with oligohydramnios and anhydramnios and the fact that Trop-2 was initially identified in placental tissues and is highly expressed on trophoblast cells, there is significant concern that ADCs targeting either HER2 or Trop 2 may pose a high risk of fetotoxicity.^52^ Thus, ADCs are currently contraindicated in pregnancy. Immunotherapy with pembrolizumab (a PD-1 inhibitor) is now routinely used for neoadjuvant treatment of triple-negative breast cancer (TNBC). However, immunotherapy has been associated with rare immune-related endocrinopathies such as primary hypogonadism, hypophysitis, or hypothyroidism, which can lead to increased maternal and fetal risks.^53^ Preclinical data evaluating the effect of very high dose immunotherapy in monkeys demonstrated increased risk of fetal growth restriction, premature delivery, and fetal death.^54^ However, human data from small cohorts, mainly in the setting of melanoma treatment, suggest that immunotherapy may be relatively safe in pregnancy and is not associated with worse fetal or maternal outcomes compared with other anticancer treatments.^55^ Overall, targeted therapies are currently contraindicated during pregnancy, and effective contraception is recommended for reproductive-age women receiving these treatments. However, as more YWBC undergo targeted therapy for early-stage disease, research into the safety of these agents during pregnancy will become increasingly important.

## Fertility and future pregnancy

Beyond the management of breast cancer during pregnancy, it is essential to understand the impact of breast cancer therapies on subsequent pregnancy outcomes. With the rising incidence of YWBC, considerations for this unique population must encompass potential effects of breast cancer treatment on long-term reproductive health and fertility. In a prospective cohort study of YWBC, 36% of women reported interest in having biological children within 5 years of diagnosis.^56^ About half (51%) of YWBC reported concerns about infertility related to breast cancer treatment. Additionally, fertility and family planning considerations influenced treatment decisions in 18% of respondents, impacting choices such as the type of chemotherapy received, the decision to undergo endocrine therapy, and the duration of endocrine therapy.^57^ This data emphasizes the importance of future fertility to YWBC. As such, standard fertility preservation strategies—including oocyte and embryo cryopreservation—should be discussed with all reproductive-age women prior to initiation of therapy. Early referral to a reproductive endocrinologist is recommended for timely counseling and intervention. Even when established fertility preservation methods such as oocyte and embryo cryopreservation are not feasible, clinicians may consider the use of gonadotropin-releasing hormone agonists as an alternative strategy to reduce the risk of chemotherapy-induced ovarian insufficiency. This approach is particularly valuable when cancer treatment must begin urgently.^58^

In addition to fertility preservation before treatment and managing the risk of ovarian insufficiency during therapy, new data may help guide clinicians in safely de-escalating treatment for patients with active plans to pursue pregnancy. This is particularly relevant in hormone receptor-positive breast cancer, where adjuvant endocrine therapy is typically recommended for 5–10 years, during which there is naturally a decline in fertility due to ovarian aging. For patients who desire future pregnancy, this prolonged treatment period is a significant burden, as pregnancy is contraindicated during endocrine therapy. Encouragingly, recent data on the safety of interrupting endocrine therapy to pursue pregnancy suggest that patients can safely pause treatment without an increased risk of recurrence. The POSITIVE trial demonstrated that temporarily interrupting endocrine therapy for up to 2 years to attempt pregnancy did not significantly increase the short-term risk of breast cancer recurrence. At a median follow-up of 41 months, the 3-year breast cancer recurrence rate for women who interrupted endocrine therapy was 8.9%, comparable to the 9.2% rate observed in external controls.^59^ The majority of participants had stage I or II disease (93.4%), and nearly two-thirds (66.2%) had lymph node-negative disease. In patients with higher-risk features—such as lymph node positivity, higher tumor grade (grade 3), and larger tumor size (>2 cm)—there was a trend toward a higher cumulative incidence of breast cancer events compared to controls; however, these subgroup analyses were not powered for statistical significance. Among participants who provided pregnancy data, 74.0% reported becoming pregnant at least once during the trial, which should be highly encouraging to young breast cancer patients. Additionally, 43.3% of participants reported using assisted reproductive technology. These findings provide important reassurance for young hormone receptor-positive breast cancer patients. The results of the POSITIVE trial demonstrate that pregnancy after endocrine therapy interruption is both feasible and, in the short term, safe, especially in those patients with lower-risk disease.

There remains a need for more robust data to help refine which subgroups of YWBC may safely interrupt treatment to pursue pregnancy. In particular, subsequent pregnancy outcomes in higher-risk subgroups, such as those with PABC, warrant further investigation. Data from a small cohort of 75 patients with PABC examined differences between those who had subsequent pregnancies and those who did not. The study found no significant differences in clinical outcomes, including disease-free survival and overall survival.^60^ However, given the association of PABC with more aggressive tumor biology, the limited sample size highlights the need for larger studies to better understand the safety of pregnancy in this population. Similarly, in a cohort study of YWBC patients with pathogenic BRCA mutations, subsequent pregnancy after a breast cancer diagnosis was not linked to adverse maternal prognosis or fetal outcomes.^61^ Overall, these findings are encouraging; however, continued research is necessary to inform individualized counseling for patients in higher-risk subgroups.

Another key question with significant implications for future fertility is whether chemotherapy can be de-escalated in YWBC. Previous studies, including TAILORx and RxPONDER, suggest that young women, unlike postmenopausal women, with Oncotype DX recurrence scores RS 16–25 (for pN0 patients age 50 years and younger) or 0–25 (for pN1 premenopausal patients) derive some benefit from chemotherapy.^62,63^ However, it remains unclear whether this benefit stems from the direct cytotoxic effects of chemotherapy or from chemotherapy-induced ovarian suppression. The OFSET trial (NRG-BR009) is a forthcoming phase III study that aims to address the critical question of whether premenopausal women with early-stage, ER-positive/HER2-negative breast cancer truly benefit from cytotoxic chemotherapy outside of its secondary effects, causing ovarian function suppression.^64^ If OFSET demonstrates that the therapeutic effect of chemotherapy in intermediate-risk premenopausal women is largely attributable to ovarian suppression, it could fundamentally shift treatment strategies. Clinicians may be able to opt for targeted ovarian suppression alone in this subset of patients, avoiding the accelerated loss of ovarian reserve associated with cytotoxic chemotherapy. The results of this trial have the potential to inform more personalized, fertility-preserving treatment strategies in YWBC.

Recent advances in systemic therapies for early-stage breast cancer—including CDK 4/6 inhibitors for ER+/HER2−, PARP inhibitors for patients with germline BRCA mutations, adjuvant ADCs for residual disease in HER2+ breast cancer, and perioperative immunotherapy such as pembrolizumab for TNBC—have advanced management for prevention of recurrent breast cancer.^65–68^ However, their effects on ovarian function and fertility in YWBC are poorly understood. CDK4/6 inhibitors play important roles in ovarian physiology, but preclinical and clinical data on their gonadotoxicity are limited and conflicting, with some evidence suggesting potential ovarian toxicity, while other studies indicate possible protective effects.^69–71^ Anti-HER2 therapies like trastuzumab and pertuzumab generally do not increase the risk of treatment-related amenorrhea (TRA).^72–74^ However, there is less data on the effect of HER2-targeted ADCs like T-DM1 on ovarian function. Encouragingly, findings from the ATEMPT trial found that T-DM1 was associated with lower rates of chemotherapy-related amenorrhea at 18 months compared to paclitaxel and trastuzumab.^75^ PARP inhibitors, particularly olaparib, have shown potential reproductive toxicity in preclinical studies, including reduced oocyte number and primordial follicle loss in mice.^76,77^ No clinical data currently exist on their impact on ovarian function or TRA in humans. Immune checkpoint inhibitors may impact ovarian function as measured by anti-Müllerian hormone (AMH) levels, which has been seen in melanoma patients.^78^ However, the decline in AMH appears less severe than that seen with chemotherapy. Overall, more research is needed to clarify the long-term effects of these targeted treatments on ovarian reserve. Notably, clinical trials are not consistently designed to collect data on the effects of investigational drugs on ovarian reserve and fertility. A recent analysis of 141 eligible neoadjuvant and adjuvant breast cancer trials found that only 4 (2.8%) included post-intervention AMH data, a surrogate marker of ovarian reserve.^79^ These findings highlight the significant gap in knowledge in this area, which demands the need for intentional trial design to address fertility outcomes in YWBC (see Table 1).

**Table 1.** Safety of systemic therapies in PrBC and for future pregnancy.

Therapy	Safety in pregnancy	Safety in breastfeeding	Effect on future pregnancy
Chemotherapy (anthracyclines, alkylating agents, taxanes)	Safe in 2nd and 3rd trimester. Contraindicated in 1st trimester due to timing of organogenesis.^42,43^	Contraindicated during active chemotherapy; drug excretion in breastmilk and risk of gut toxicity to infant.^80^	May impair ovarian reserve, especially alkylating agents; however, over 60% of women aged 40 years and younger recover ovarian function in 1 year.^74^
HER2-targeted therapies (e.g., trastuzumab, pertuzumab)	Contraindicated. Associated with oligohydramnios and anhydramnios.^47,81^	Not recommended. Insufficient data, likely minimally excreted in breast milk.^80^	No increase in risk of TRA.^72,73^
Endocrine therapy (e.g., tamoxifen)	Contraindicated. Linked to increased major fetal malformations.^74^	Contraindicated. Excreted in breastmilk and accumulates over time.^80^	Reversible effect on fertility^74^
Immunotherapy (e.g., pembrolizumab)	Avoid use. Rare endocrine complications (e.g., hypophysitis). Animal data suggest fetal risks; human data inconclusive.^53–55^	Not recommended; Insufficient data, likely minimally excreted in breast milk.^80^	Preclinical data suggest reduced oocyte number and quality in mice. Immunotherapy may cause a decrease in AMH levels, but less severe than with chemotherapy.^78,82^
CDK4/6 inhibitors (e.g., palbociclib and ribociclib)	Contraindicated. Animal studies show embryotoxicity; interference with cell cycle.^49^	Not recommended. Insufficient data, likely minimally excreted in breast milk.^80^	Preclinical data are conflicting. Needs more research.^69–71^
PARP inhibitors (e.g., olaparib)	Contraindicated. Teratogenic in animal models; interfere with DNA repair.^51^	Not recommended. Insufficient data, likely minimally excreted in breast milk.^80^	Preclinical data suggest reduced oocyte number in mice. Needs more research.^76,77^
ADCs (e.g., T-DM1, trastuzumab deruxtecan, sacituzumab govitecan)	Contraindicated. Concerns due to fetal expression of Trop-2 (placental protein) and HER2 toxicity.^52^	Not recommended. Insufficient data, given toxic payload, there is potential for adverse reactions in breastfed infant.^80^	Lower rates of TRA in T-DM1 compared to systemic chemotherapy. Needs more research.^75^

ADCs, antibody drug conjugates; AMH, anti-Müllerian hormone; CDK4/6, cyclin-dependent kinases 4/6; PARP, poly-ADP-ribose polymerase; PrBC, pregnancy-related breast cancer; T-DM1, trastuzumab emtansine; TRA, treatment-related amenorrhea.

It is important to consider the effects of local therapies on subsequent pregnancies and breastfeeding. Breastfeeding after breast-conserving surgery and radiation is possible, although milk production may be severely or completely reduced in the treated breast. For women who have undergone a mastectomy, breastfeeding from the contralateral breast also remains an option. The POSITIVE study provided encouraging data on breastfeeding after breast cancer treatment, showing that 63% of patients who had a live birth and did not have a bilateral mastectomy were able to breastfeed, with 37% breastfeeding for at least 6 months. Factors associated with a higher likelihood of breastfeeding included being older (⩾35 years), having no prior children, and undergoing breast-conserving surgery. Importantly, breastfeeding did not impact the breast cancer-free interval at 2 years, although longer follow-up is still needed.^83^ Similar findings from a recently published prospective cohort study of patients with YWBC had similar findings: most patients with pregnancies after breast cancer without bilateral mastectomy who attempted to breastfeed were able to and were satisfied with their breastfeeding experience.^84^ These findings suggest that breastfeeding after breast cancer treatment is feasible and does not appear to compromise cancer outcomes.

Fertility considerations for YWBC should be guided by early, multidisciplinary planning. All reproductive-age patients should receive timely education before initiating treatment regarding the potential impact of cancer therapies on fertility. Early referral to a reproductive endocrinologist is strongly recommended to facilitate personalized fertility preservation strategies or interventions aimed at reducing the risk of treatment-induced ovarian insufficiency. For patients with hormone receptor-positive disease who desire future pregnancy, emerging data support the safety of interrupting endocrine therapy after 18–24 months in carefully selected patients with lower-risk disease. Patients should be counseled that endocrine therapy must be discontinued for a full 3 months prior to attempting conception. Looking ahead, results from the OFSET trial may provide critical insights into which premenopausal patients truly benefit from chemotherapy, ultimately guiding more tailored treatment decisions that also take fertility preservation into account. Providing optimal fertility care to YWBC requires a coordinated, multidisciplinary approach. In addition to medical oncologists and reproductive endocrinologists, care teams should include breast surgeons, who play a key role in surgical decision-making with potential implications for future breastfeeding. For instance, in women with future breastfeeding goals, breast-conserving surgery may offer advantages over mastectomy when clinically appropriate. Psychosocial support providers are also critical members of the care team, as emphasized by the American Society of Clinical Oncology guidelines, which recommend inclusion of counseling services to address the emotional and psychological impact of fertility-related decisions. Ultimately, fertility care in YWBC should be individualized and patient-centered, informed by the patient’s personal preferences, reproductive goals, and clinical risk profile.

## Conclusion

The relationship between pregnancy, its influence on breast cancer risk, and the subsequent impact of breast cancer on future pregnancies in young women is complex. Further research is needed to elucidate the unique biology of PPBC, to develop screening and prevention strategies for those at risk, and to optimize treatment and surveillance approaches in YWBC. Meanwhile, breast cancer treatment is evolving rapidly, with newer targeted therapies being introduced in the early-stage setting. Significant gaps remain in understanding these therapies—particularly their safety during pregnancy and nursing, impact on fertility, and effects on future pregnancies. Clinical trials should prioritize the concerns of YWBC by incorporating fertility outcomes into drug development and assessment. In addition, research into the psychosocial and quality-of-life aspects of young breast cancer survivors navigating fertility and family planning decisions is essential. These studies will provide critical insights to improve patient counseling, guide treatment decisions, and ultimately enhance the comprehensive care of YWBC, both during and after pregnancy.

## Declarations

**Ethics approval and consent to participate:** Not applicable.

**Consent for publication:** Not applicable.

**Availability of data and materials:** Not applicable.
